# A mass in the upper abdomen derived from *Talaromyces marneffei* infected lymphadenopathy: a case report

**DOI:** 10.1186/s12879-021-06489-7

**Published:** 2021-08-04

**Authors:** Xue Chen, Lin Jia, Yongfeng Wu, Jing Chang, Tong Zhang, Yingmin Ma, Yulin Zhang

**Affiliations:** 1 Department of Respiratory and Infectious Diseases, Beijing You An Hospital, Capital Medical University, Beijing Institute of Hepatology, Beijing, 100069 China; 2 Pathology Diagnostic Center, Beijing You An Hospital, Capital Medical University, Beijing Institute of Hepatology, Beijing, 100069 China

**Keywords:** *Talaromyces marneffei*, HIV, AIDS, Upper abdominal mass, Next-generation sequencing

## Abstract

**Background:**

An upper abdominal mass without tenderness often indicates a benign or malignant tumor once liver or spleen hyperplasia has been excluded. A lymphadenopathic mass from *Talaromyces marneffei* infection is rare.

**Case presentation:**

We report the case of a 29-year-old human immunodeficiency virus (HIV) infected man who presented with an upper abdominal mass and without any symptoms related with infection. Histopathology and next-generation sequencing (NGS) following biopsy of the mass confirmed *T. marneffei*-infected lymphadenopathy, and the patient was successfully treated with amphotericin B and itraconazole.

**Conclusions:**

This case report suggests that potential fungal infection should be considered during the diagnostic workup of a mass in clinical practice.

## Background

Upper abdominal distension, along with a non-tender mass, often suggests a benign or malignant tumor once liver or spleen hyperplasia has been excluded. A series of imaging and pathologic examinations are often required to diagnose the mass. However, an upper abdominal mass due to fungal lymphadenopathy is rare. Talaromycosis caused by *Talaromyces marneffei* is a regional fungal disease that is endemic to Southeast Asia, India, and southern China [1, 2]. Talaromycosis is a life-threatening mycosis that primarily affects immunocompromised individuals and it is common in people living with HIV and is more likely to spread through the blood and affect the whole body [1]. As one of the acquired immunodeficiency syndrome (AIDS)-defining diseases, the mortality and morbidity rates of talaromycosis are preceded only by HIV-related tuberculosis and cryptococcosis in Thailand [3]. Although the incidence of talaromycosis in people living with HIV has decreased because of the widespread use of antiretroviral therapy, its mortality is still as high as 20% [4]. The infection usually starts as a subacute disease: patients commonly develop fever, weight loss, hepatosplenomegaly, lymphadenopathy, and abnormal symptoms of respiratory and gastrointestinal diseases [5]. Skin bumps with a small dent in the center are a common manifestation, in addition to fever and other infection-related presentations [6]. Herein, we describe the diagnostic workup for a patient with an upper abdominal mass derived from *T. marneffei*-infected lymphadenopathy.

## Case presentation

**Fig. 1:**
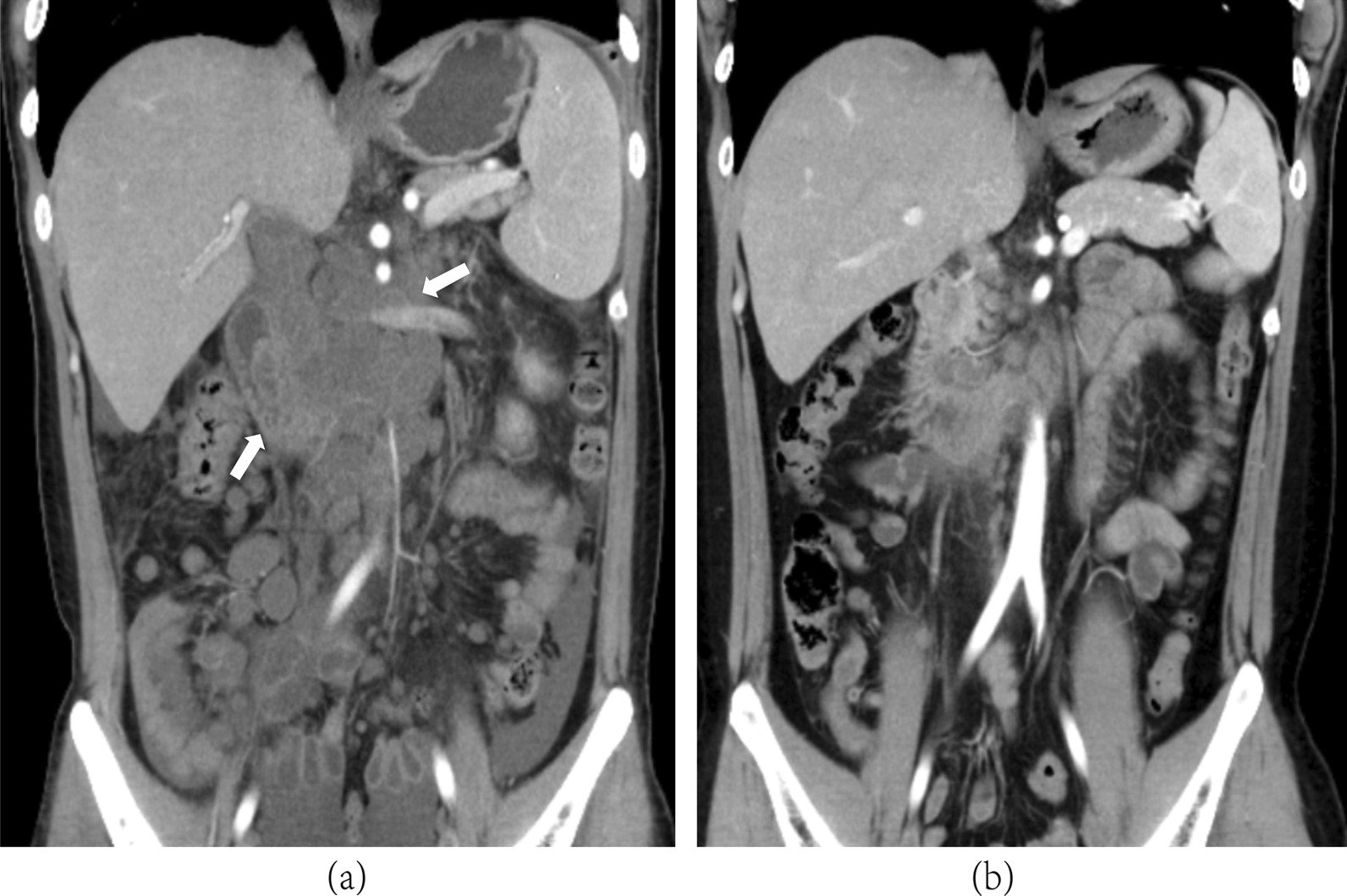
Abdominal CT scan (**a**) shows a giant mass in the upper abdomen, which demonstrated shrinkage after treatment (**b**)

**Fig. 2:**
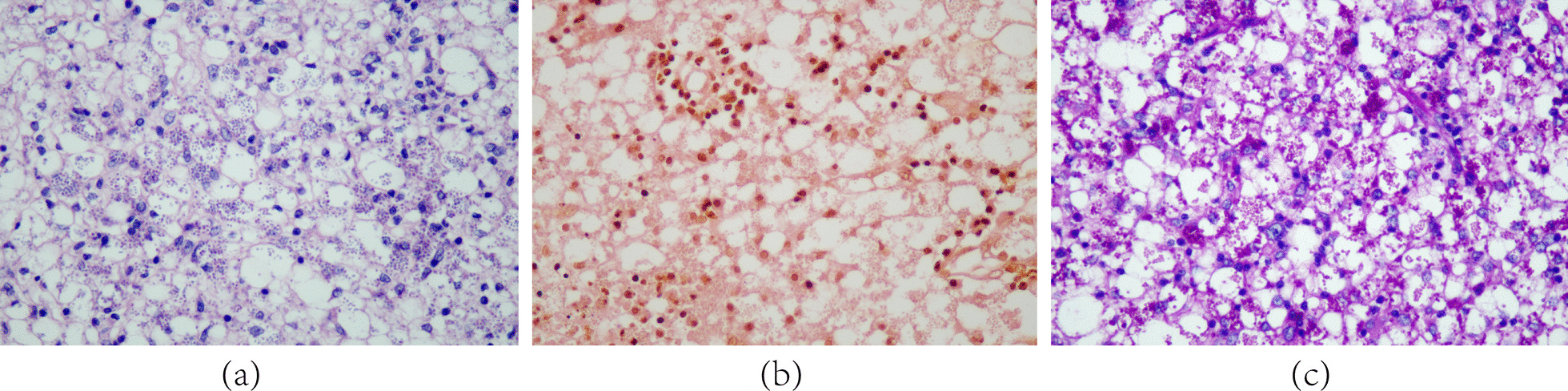
Hematoxylin–eosin staining (**a** magnification, ×400), Gomori methenamine silver staining (**b** magnification, ×400), and periodic acid schiff staining (**c** magnification, ×400), show large areas of soft tissue necrosis in the cervical lymph nodes accompanied with a large number of foam cell reactions around them, along with multiple yeast-like cells gathered in the cytoplasm, suggesting a fungal infection

**Fig. 3:**
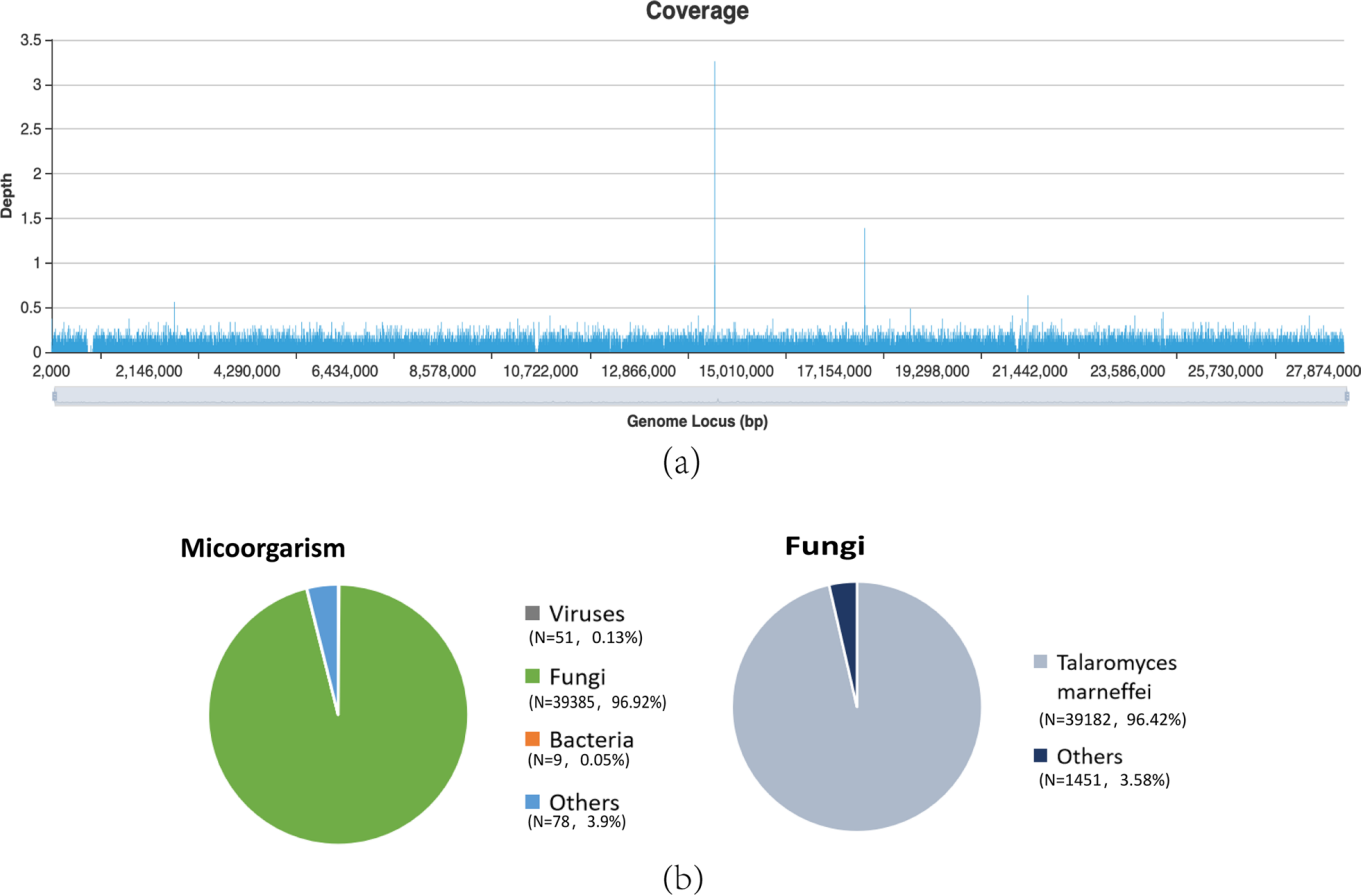
NGS coverage map of tissue from the retroperitoneal lymph nodes suggested that *T. marneffei* was the main pathogen, and a total coverage of 9.37% was obtained (**a**). Distribution of viruses, bacteria, and fungi identified in the retroperitoneal lymph node tissue by NGS revealed that *T. marneffei* was the main pathogen, with a sequence number of 39,182 and highest relative abundance of 96.42% (**b**)

A 29-year-old man presented with abdominal distension, which had lasted for more than 2 months, accompanied by a weight loss of 10 kg. No complaints of chills and fever, nausea, vomiting, abdominal pain, or diarrhea were reported by this patient. Physical examination revealed a swollen abdomen with a mass of 10 cm in diameter in the upper abdomen and multiple swollen lymph nodes in the neck. HIV antibody test was positive, and HIV-1 infection was confirmed with only 8 CD4^+^ cells/μL. Laboratory tests revealed that he had anemia and leukopenia, in addition to elevated erythrocyte sedimentation rate, C-reactive protein and procalcitonin levels (Table 1). Abdominal computed tomography (CT) scan showed a giant mass in the upper abdomen (Fig. 1a). Based on the clinical and imaging findings, lymphoma was suspected. Subsequently, a cervical lymph node biopsy was performed, which demonstrated large areas of tissue necrosis in the lymph nodes, along with a large number of foam cell reactions around them as well as numerous yeast-like cells in the cytoplasm, suggesting a fungal infection (Fig. 2). The *T. marneffei* infection was initially diagnosed by NGS of the retroperitoneal lymph node tissue, which revealed a *T. marneffei* fungemia of 39,185 unique reads with 96.42% coverage of identified fungal genes in 72 h (Fig. 3). The blood and tissue culture grew *T. marneffei* in 11 and 5 days, respectively, confirming the diagnosis of *T. marneffei* infection.

**Table 1:** Laboratory test results on admission

Test item	Test value	Normal range
White blood cell counts (10^9^/L)	2.44	3.5–9.5
Neutrophils percentage (%)	80.8	40–75
Red blood cell counts(10^12^/L)	3.5	4.3–5.8
Hemoglobin (g/L)	81.0	130–175
Platelets (10^9^/L)	269	125–350
Blood urea nitrogen (mmol/L)	4.14	2.29–7.0
Creatinine (μmol/L)	57	53–106
Alanine transarninase (U/L)	45	9–50
Glutamic-oxal acetic transaminase (U/L)	80	15–40
Total protein (g/L)	63.7	65–85
Albumin (g/L)	27.9	40–55
Creatine kinase (U/L)	34	50–310
Lactate dehydrogenase (U/L)	288	120–250
CD4 cell counts (cells/μL)	8	600–800
Erythrocyte sedimentation rate (mm/h)	85	< 15
High-sensitivity C-reactive protein (mg/L)	78.6	0–3
Procalcitonin (ng/mL)	0.21	< 0.1
Plasma (1,3) beta-d-glucan (pg/mL)	72.1	< 60
Serum galactomannan antigen	Negative	Negative
Cryptococcus antigen	Negative	Negative
Anti-EBV-EA immunoglobulin M antibody	Negative	Negative
Anti-EBV-VCA immunoglobulin M antibody	Negative	Negative
Anti-CMV immunoglobulin M antibody	Negative	Negative
Anti-CMV immunoglobulin G antibody	Positive	Negative
Anti-TOX immunoglobulin M antibody	Negative	Negative
Anti-TOX immunoglobulin G antibody	Negative	Negative
EBV DNA (copies/mL)	< 500	< 500
CMV DNA (copies/mL)	2.33 * 10^3^	< 500
HIV RNA loads(copies/mL)	10 × 10^6^	< 500

*EBV* Epstein–Barr virus, *CMV* cytomegalo virus, *EA* early antigen, *VCA* viral capsid antigen, *HIV* human immunodeficiency virus, *Tox* toxoplasma

After the diagnosis was considered to be a fungal infection following neck lymph node biopsy, treatment with intravenous amphotericin B was initiated. When the diagnosis of *T. marneffei* was confirmed by NGS, the current treatment was continued. After 2 weeks, oral itraconazole for consolidation therapy along with antiretroviral therapy was started, resulting in a favorable clinical response in 1 month and shrinkage of the abdominal mass (Fig. 1b). The patient was discharged from the hospital in a stable condition.

## Discussion and conclusions

Lymphadenopathy is extremely common in patients with HIV infection and has several possible causes, including the generalized lymphadenopathy of HIV-infection, malignancy, and single or multiple co-infections [7]. As with the case that we reported, HIV-infected person with abdominal lymphadenopathy as the main clinical symptom may be finally diagnosed with *T. marneffei* infection. *T. marneffei* can cause infection in immunocompromised individuals with, or without, a history of residence in, or travel to, an endemic region [8]. It is the most important thermally dimorphic fungus, which can cause respiratory, skin, and systemic mycosis in China and Southeast Asia [9]; it is also a life-threatening mycosis that primarily affects immunocompromised individuals [1]. The main risk factor for *T. marneffei* infection is cell-mediated immune dysfunction, which is usually secondary to HIV infection, especially in people with CD4^+^ cell counts below 100 cells/μL [10]. The symptoms of *T. marneffei* infection vary. The most common symptoms include fever, weight loss, malaise and anemia. There may also be fungemia, lymphadenopathy, hepatomegaly, lung disease (non-productive cough and dyspnea), diarrhea, splenomegaly, and systemic skin lesions [11]. Skin lesions are present in 60–70% of patients, which are often the early manifestations of disseminated cases, and more common on the face, trunk and upper limbs [6]. The common laboratory findings of *T. marneffei* infection include anemia, thrombocytopenia, and elevated transaminases [12]. The laboratory examination results of the patient we reported suggest anemia and leukopenia in addition to elevated erythrocyte sedimentation rate, C-reactive protein, and procalcitonin levels. These were helpful in making the early diagnosis of *T. marneffei* infection.

Microscopic findings of intramacrophage and extramacrophage yeast organisms in smears of skin lesions, lymph nodes, and bone marrow aspirate can lead to a rapid presumptive diagnosis [6]. In addition, *T. marneffei* is a dimorphic fungus, and a diffusible red pigment that can be seen on culture can confirm the diagnosis. However, the very long time for culture, can lead to delays in diagnosis and increased mortality, especially in patients without skin lesions [8]. The histopathologic finding of intracellular and extracellular yeast forms and the characteristic cross-septation of *T. marneffei* highlighted by Gomori methenamine silver staining from specimens obtained from the affected tissue or *T. marneffei* grew in cultures that could identified the diagnosis [13]. In atypical and severe cases, it is difficult to establish a rapid diagnosis using traditional assays. The patient whose case we reported, was characterized mainly with an abdominal mass, but without the typical *T. marneffei* symptoms, such as skin lesions. Finally, the nucleotide sequence of *T. marneffei* in the abdominal mass samples from our patient, was identified quickly by NGS. It can be seen that NGS testing brings the dawn of diagnosis detect rare pathogen and takes a shorter time [14]. Actually, The current gold standard for the diagnosis of *T. marneffei* infection is pathogen culture, but culture generally takes 7–10 days, making early diagnosis difficult. In contrast, NGS diagnosis only takes 24–72 h [15].

Antifungal therapy is the first choice of treatment for *T. marneffei*, and it is classified into induction, consolidation, and maintenance stages. International guidelines recommend the use of amphotericin B deoxycholate for initial (induction) treatment at a dose of 0.7 to 1 mg per kilogram of body weight per day for 2 weeks, followed by itraconazole at a daily dose of 400 mg for 10 weeks [16]. A recent randomized controlled trial on itraconazole versus amphotericin B in the treatment of penicilliosis in Vietnam suggests that amphotericin B is superior to itraconazole at induction stage in HIV related *T. marneffei* infection [17].

Unlike previously reported cases, we reported an unusual case of *T. marneffei* infection in a patient with AIDS, the principal clinical manifestations of abdominal distension and abdominal mass. Due to the lack of specific clinical manifestations, *T. marneffei* infection could likely be misdiagnosed as tuberculosis, histoplasmosis, cryptococcosis, and lymphoma in patients with systemic lymphadenopathy. Accordingly, it is necessary to emphasize the need to diagnose this disease. In the case of atypical talaromycosis, the diagnosis is more rapid by NGS than by biopsy or culture, allowing rapid initiation of therapy, which is particularly important in immunocompromised patients. Hence, rapid diagnosis is of significant benefit to these patients.

## Data Availability

All data generated or analyzed during this study are included in this published article. The data that support the findings of this study are available from the corresponding author upon reasonable request.

## Abbreviations

HIV: Human immunodeficiency virus
AIDS: Acquired immunodeficiency syndrome
CT: Computed tomography
NGS: Next-generation sequencing
